# C-Terminal *Clostridium perfringens* Enterotoxin-Mediated Antigen Delivery for Nasal Pneumococcal Vaccine

**DOI:** 10.1371/journal.pone.0126352

**Published:** 2015-05-27

**Authors:** Hidehiko Suzuki, Akihiro Watari, Eri Hashimoto, Miki Yonemitsu, Hiroshi Kiyono, Kiyohito Yagi, Masuo Kondoh, Jun Kunisawa

**Affiliations:** 1 Laboratory of Vaccine Materials, National Institute of Biomedical Innovation, Health and Nutrition, Osaka 567–0085, Japan; 2 Laboratory of Bio-Functional Molecular Chemistry, Graduate School of Pharmaceutical Sciences, Osaka University, Suita, Osaka 565–0871, Japan; 3 Division of Mucosal Immunology, Department of Microbiology and Immunology, The Institute of Medical Sciences, The University of Tokyo, Tokyo 108–8639, Japan; 4 International Research and Development Center for Mucosal Vaccines, The Institute of Medical Science, The University of Tokyo, Tokyo 108–8639, Japan; 5 Core Research for Evolutional Science and Technology (CREST), Japan Science and Technology Agency, Tokyo, Japan; 6 Department of Microbiology and Infectious Diseases, Kobe University Graduate School of Medicine, Kobe 650–0017, Japan; 7 Graduate School of Medicine, Graduate School of Pharmaceutical Sciences, and Graduate School of Dentistry, Osaka University, Osaka 565–0871, Japan; Instituto Butantan, BRAZIL

## Abstract

Efficient vaccine delivery to mucosal tissues including mucosa-associated lymphoid tissues is essential for the development of mucosal vaccine. We previously reported that claudin-4 was highly expressed on the epithelium of nasopharynx-associated lymphoid tissue (NALT) and thus claudin-4-targeting using C-terminal fragment of *Clostridium perfringens* enterotoxin (C-CPE) effectively delivered fused antigen to NALT and consequently induced antigen-specific immune responses. In this study, we applied the C-CPE-based vaccine delivery system to develop a nasal pneumococcal vaccine. We fused C-CPE with pneumococcal surface protein A (PspA), an important antigen for the induction of protective immunity against *Streptococcus pneumoniae* infection, (PspA-C-CPE). PspA-C-CPE binds to claudin-4 and thus efficiently attaches to NALT epithelium, including antigen-sampling M cells. Nasal immunization with PspA-C-CPE induced PspA-specific IgG in the serum and bronchoalveolar lavage fluid (BALF) as well as IgA in the nasal wash and BALF. These immune responses were sufficient to protect against pneumococcal infection. These results suggest that C-CPE is an efficient vaccine delivery system for the development of nasal vaccines against pneumococcal infection.

## Introduction

Because various pathogens infect through mucosal tissues, the induction of protective immunity at mucosal tissues is a primary strategy to prevent infectious diseases. In vaccine development, injection-based immunization induces systemic immune responses but not mucosal immune responses and so fails to prevent invasion of pathogens at mucosal sites. In contrast, mucosal vaccines (e.g., nasal and oral vaccine) induce both systemic and mucosal immune responses [1]. Therefore, mucosal vaccines have been considered to be ideal for the prevention of and protection from infectious diseases. It is generally accepted that the development of an effective and safe vaccine delivery system is essential for the development of mucosal vaccine against respiratory and intestinal infectious diseases.

Mucosa associated-lymphoid tissues (MALTs) play a pivotal role in the induction of antigen-specific immune responses against mucosally administered antigens, since the tissues have been shown to contain all the necessary immunocompetent cells for the initiation of antigen-specific immune response [2–4]. Therefore, the delivery of antigen to MALT is a promising approach for the development of mucosal vaccine [5, 6]. A primary target of vaccine delivery is MALT epithelium, where M cells are located and play an important role in antigen uptake from the lumen and transport into MALTs [3]. Targeting M cells by using specific antibodies [7, 8] and bacterial invasion molecules [9] as vaccine delivery vehicle efficiently deposited antigen to MALT and induced immune responses against conjugated antigens. Another target is epithelial cells, which cover the entire mucosal tissues and form tight junctions to seal off the intercellular space. Tight junctions are composed of claudin, occludin, tricellulin, and zonula occludens [10]. There are more than 20 members of the claudin family, whose expression profiles and functions differ among tissues.

We previously found that claudin-4 was highly expressed in nasopharynx-associated lymphoid tissue (NALT) [11] and thus targeting caludin-4 would be a logical delivery candidate for a nasal vaccine. To this end, we used C-terminal fragment of *Clostridium perfringens* enterotoxin (C-CPE), a non-toxic element of *Clostridium perfringens* (CPE) that binds to claudin-4 [12, 13]. Our previous study showed that intranasal immunization of ovalbumin (OVA) fused C-CPE induced OVA-specific systemic and mucosal immune responses by claudin-4 binding of C-CPE [11, 14]. These findings allow us to examine whether claudin-4-targeting vaccines using C-CPE were effective for generating mucosal vaccines against infectious diseases.

*Streptococcus pneumoniae* (*S*. *pneumoniae*) is a key respiratory pathogen and causes pneumonia, meningitis, and otitis media [15, 16], which are calassfied more than 90 serotype [17, 18]. Polysaccharide-based injection-type vaccines are currently used as pneumococcal vaccines and success to reduce the incident of pneumococcal disease [19, 20]. However, the effect of these polysaccharide-based injection-type vaccines are only induced serotype specific immune responses. Thus, they do not cover all stains of *S*. *pneumoniae* and thus are ineffective for unrelated strains. Therefore, it is necessary to develop a pneumococcal vaccine which is effective for all strains of *S*. *pneumoniae*. Pneumococcal surface protein A (PspA) is a choline-binding surface protein of *S*. *pneumoniae* and protects *S*. *pneumoniae* from killing by apolactoferrin [21]. PspA has high antigenicity, is expressed on all isolates of *S*. *pneumoniae* [22]. Additionally, PspA induces cross-reactivity among different strains [23]. Moerover, PspA induces cross active immune responses not only in mice but also in human [23, 24]. Thus, PspA is considered to be an ideal vaccine antigen for the development of a pneumococcal nasal vaccine. In this study, we used C-CPE as a nasal delivery vehicle of PspA to create a nasal vaccine against pneumococcal infection.

## Materials and Methods

### Mice

Female BALB/c mice (age, 6 to 7 weeks) were purchased from SLC, Inc. (Shizuoka, Japan). In some experiments, we checked murine condition at least once per day. Since mice havindg 30% of body weight loss would lead to death soon, we monitored body weighy everyday. We killed mice if they reach to 30% reductoin in their body weight or after 14 days after infection. All experiments were approved by the Animal Care and Use Committee of Graduate School of Pharmaceutical Sciences, Osaka University (#22-7-0) and the Animal Care and Use Committee of the National Institute of Biomedical Innovation (approved # DS25-3R4), and conducted in accordance with their guidelines.

### Cell culture

A mouse fibroblast cell line (L cells) and mouse claudin-4-expressing L cells were kindly provided by Dr. S. Tsukita (Kyoto University, Kyoto, Japan) [12]. L cells and claudin-4-expressing L cells were cultured in modified Eagle’s medium supplemented with 10% fetal bovine serum in a 5% CO_2_ atmosphere at 37°C.

### Preparation of PspA-C-CPE fusion protein

PspA cDNA was amplified by polymerase chain reaction (PCR) amplification (forward primer: 5′-agggtaccgaagaatctcccgtagcc-3′, *Kpn*І site is underlined; reverse primer: 5′-gcttaattaattctggggctggagtttc-3′ *Pac*І site is underlined). pET16b-OVA-C-CPE, a G4S linker was inserted between OVA and C-CPE [11], and PspA PCR products were digested by using *Kpn*І and *Pac*І. The PspA fragment was inserted into pET-16b-C-CPE to yield pET16b-PspA-C-CPE. We also prepared PspA fragment for pET16b-PspA by PCR amplification of PspA cDNA using different primers (forward primer: 5′-atgatgatgcatatggaagaatctcccgtagcc-3′, *Nde*І site is underlined; reverse primer: 5′-gcgggatccttattctggggctggagtttc-3′ *Bam*HІ site is underlined). pET16b (Novagen, Darmstadt, Germany) and PspA PCR products were digested by using *Nde*І and *Bam*HІ. The resulting PspA fragment was inserted into pET16b to yield pET16b-PspA.

To obtain recombinant protein, the plasmids were transformed into *Escherichia coli* strain BL21 (DE3). Protein production was induced by using isopropyl-D-thiogalactopyranoside. The culture pellets were sonicated in buffer A (10 mM Tris-HCl [pH8.0], 400 mM NaCl, 5 mM MgCl_2_, 0.1 mM PMSF, 1 mM 2-mercaptoethanol, and 10% glycerol). After centrifugation, the supernatants were loaded onto HiTrap HP (GE Healthcare, Pittsburgh, PA, USA) columns. PspA or PspA-C-CPE was eluted with buffer A containing 100~500 mM imidazole. The eluted protein was loaded into a PD-10 column (GE Healthcare) for exchange with phosphate-buffered saline (PBS). The concentration of purified protein was measured by using a BCA protein assay kit (Pierce Chemical, Rockford, IL). The purity of the eluted protein was confirmed by using the NuPAGE electrophoresis system (Life Technologies, Carlsbad, California, USA) followed by staining with Coomassie brilliant blue.

### Flow cytometric analysis

Claudin-4-expressing L cells were incubated with PspA or PspA-C-CPE for 1 h at 4°C. The cells were washed with 0.1% bovine serum albumin (BSA) in PBS and incubated with mouse anti-His tag antibody (clone 13/45/31-2, mouse IgG1, Pierce) for 1 h at 4°C. After being washed with 0.1% BSA in PBS, the cells were incubated with fluorescein-labeled goat anti-mouse IgG (H+L) antibody (Rockland, Gilbertsville, PA, USA) for 30 min at 4°C. The cells were washed with 0.1% BSA in PBS and analyzed by flow cytometry (FACSCalibur, Becton Dickinson, New Jersey, USA).

For the intracellular cytokine analysis, mononuclear cells were isolated from the lung and nasal passages as previously reported [25, 26]. The isolated cells were incubated in RPMI1640 medium containing 10% fetal calf serum with 50 ng/mL of phorbol 12-myristate 13-scetate (Sigma-Aldrich, St Louis, MO), 750 ng/mL of ionomycin (Sigma-Aldrich), and 5 μg/ml of brefeldin A (BioLegend, San Diego, CA) for 4 h at 37°C. After washing with PBS, cells were stained with zombie (BioLegend) for 15 min at room temperature and subsequently treated with anti-mouse CD16/32 (clone 93, BioLegend) for 15 min at roofem temperature. After washing with PBS containing 2% newborn calf serum, the cells were staind with fluorescein isothiocyanate-rat anti-mouse CD4 (clone RM4-5, BioLegend) for 30 min at 4°C, fixed with 4% paraformaldehyde for 15 min at 4°C, permeabilized with Perm/Wash (BD Biosciences, San Diego, CA), and then stained with phycoerythrin-rat anti-mouse IL-17 (clone TC11-18H10, BD Biosciences) for 30 min at 4°C as previously reported. The cells were analyzed by flow cytometry (Miltenyi Biotec, Auburn, CA).

### Immunohistologic analysis

NALT from mice was embedded in Tissue-Tek OCT compound (Sakura Finetek Japan, Tokyo, Japan) and cut into 6-μm sections. Sections were fixed with 100% acetone for 1 min at 4°C, and nonspecific binding was blocked by treating with 2% fetal calf serum in PBS for 30 min at room temperature. Sections were washed with 0.05% Tween in PBS and incubated with PspA or PspA-C-CPE (both were biotinylated by using a biotinylation kit from Thermo Fisher Scientific (Massachusetts, USA)) and fluorescein-conjugated *Ulex europaeus* agglutinin 1 (UEA-1) at 4°C overnight. After being washed with 0.05% Tween in PBS, sections were stained with Alexa Fluor 546-conjugated streptavidin for 30 min at room temperature. Sections were washed with 0.05% Tween in PBS and stained with 4',6-diamidino-2-phenylindole (DAPI). Finally, sections were washed with 0.05% Tween in PBS, mounted in Fluoromount (Diagnostic BioSystems, California, USA), and observed by fluorescence microscopy (BZ-9000, Keyence, Osaka, Japan).

### Immunization

Mice were nasally immunized with vehicle (PBS), 5 μg of PspA alone, 2 μg of C-CPE alone or PspA-C-CPE once weekly for 3 consecutive weeks. One week after the last immunization, serum, nasal wash fluid, and bronchoalveolar lavage fluid (BALF) were collected. Nasal wash fluid was obtained by using 200 μL PBS. BALF were collected by using 1 mL PBS.

### Measurement of PspA-specific antibody production by enzyme-linked immune sorbent assay (ELISA)

PspA-specific antibody production was determined by ELISA. Accordingly, 96-well immunoplates were coated with PspA (0.05 μg/well) at 4°C overnight. The immunoplates were treated with 1% BSA in PBS for 2 h at room temperature to prevent nonspecific binding. After the plates were washed with 0.05% Tween in PBS, 2-fold serial dilutions of samples were added to wells, and the plates were incubated at 4°C overnight. After the plates were washed with 0.05% Tween in PBS, goat anti-mouse IgG or IgA conjugated with horseradish peroxidase (SouthernBiotech, Birmingham, AL) was added to the immunoplates and incubated for 1 h at room temperature. After the plates were washed with 0.05% Tween in PBS, PspA-specific antibodies were detected by using TMB peroxidase substrate and reading the absorbance at 450 nm.

### *S*. *pneumoniae* culture and infection

*S*. *pneumoniae* Xen10 (parental strain, A66.1 serotype 3; Caliper Life Sciences) were growth in brain–heart infusion broth at 37°C in a 5% CO_2_ atmosphere, with no aeration. *S*. *pneumoniae* Xen10 cells were washed and diluted with D-PBS. One week after the last immunization, mice were nasally challenged with 5.0 × 10^6^ CFU of *S*. *pneumoniae* Xen10. The survival of mice was monitored for 14 days.

### Data analysis

Data were expressed as the mean ± SEM. Statistical analysis was performed by using the non-parametric Mann–Whitney’s U test. (GraphPad Software, California)

## Results

### Preparation of PspA-fused C-CPE protein

**Fig 1:**
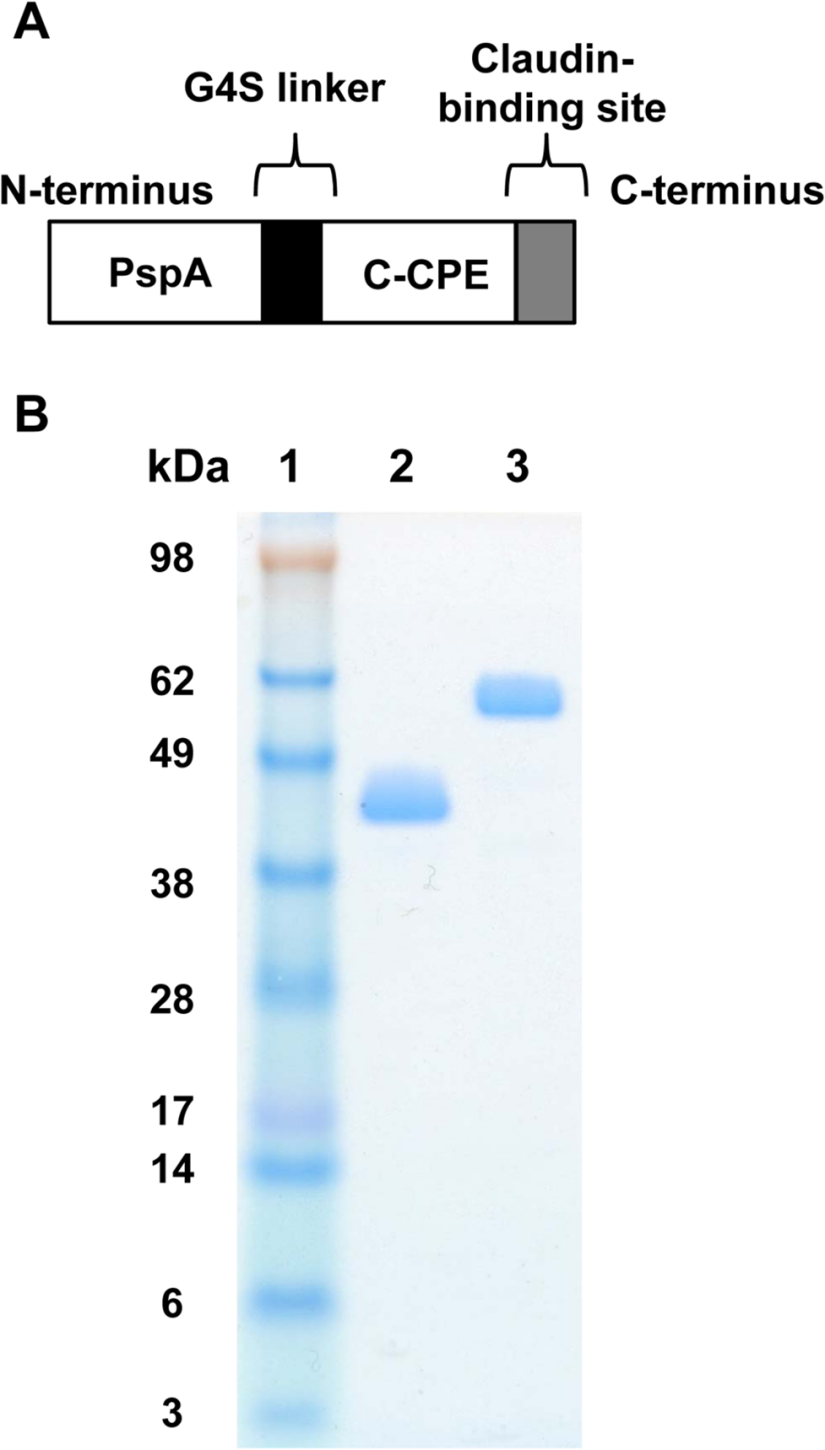
Construction and preparation of PspA-C-CPE. (A) Schematic illustration of PspA-C-CPE. PspA was fused with C-CPE at its N-terminus. A G4S linker was inserted between PspA and C-CPE. (B) PspA and PspA-C-CPE were expressed in *Escherichia coli* as His-tagged proteins and purified by Ni-affinity chromatography. The PspA and PspA-C-CPE recombinant protein were applied to SDS-PAGE followed by staining with Coomassie brilliant blue. Lane 1, size ladder; lane 2, PspA; lane 3, PspA-C-CPE.

To investigate whether a C-CPE based claudin-4-targeting vaccine delivery system can be used as a nasal pneumococcal vaccine, we genetically fused PspA with C-CPE (PspA-C-CPE). We previously found that C-terminus of C-CPE is an activite portion to interacti with claudin-4 [27]. Thus, we fused PspA with N-terminus of C-CPE to maintain the claudin-4-binding activity of C-CPE (Fig 1A). Purification of PspA and PspA-C-CPE proteins was confirmed by Coomassie brilliant blue staining (Fig 1B).

**Fig 2:**
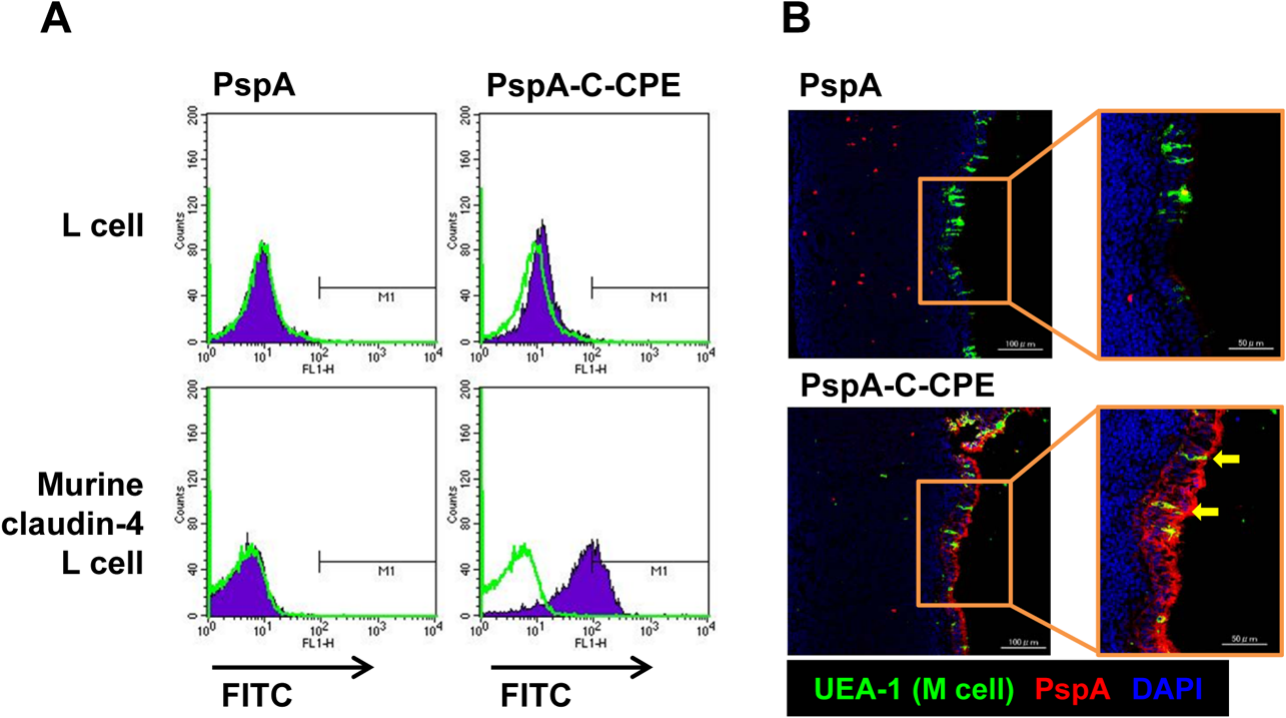
Binding of PspA-C-CPE to claudin-4-expressing cells. (A) Claudin-4-expressing L cells and parent L cells were treated with PspA or PspA-C-CPE. Their bindings were detected by using an anti-His tag antibody followed by staining with fluorescein-labeled secondary antibody. Violet histograms are PspA or PspA-C-CPE; the green line histogram is control. (B) Binding of PspA-C-CPE to NALT epithelium. NALT sections were fixed with acetone and stained with biotinylated-PspA or biotinylated-PspA-C-CPE followed by staining with Alexa Fluor 546-conjugated streptavidin. M cells were detected by staining with fluorescein-conjugated UEA-1. Yellow arrows indicate PspA-C-CPE bound to M cells. Red, biotinylated-PspA or biotinylated-PspA-C-CPE; green, UEA-1; blue, DAPI. Scale bar is 100 μm (left) or 50 μm (right).

We then checked the binding activity of PspA-C-CPE to claudin-4. We previously reported that OVA-fused with C-CPE bound to claudin-4 [11]. Likewise, PspA-C-CPE efficiently bound to claudin-4-expressing L cells but not to parent L cells (Fig 2A). We also confirmed that PspA alone did not bind to L cells regardless of whether they expressed claudin-4 (Fig 2A).

We next investigated whether PspA-C-CPE bound to NALT epithelium. As seen for the binding of PspA-C-CPE to claudin-4-epxressing L cells, PspA alone did not bind to NALT epithelium, whereas the PspA-C-CPE construct efficiently bound to epithelium (Fig 2B). Additionally, PspA-C-CPE also bound to UEA-1^+^ M cells, a finding consistent with a previous report that M cells also expressed claudin-4 [28]. These data indicate that PspA-C-CPE maintained binding activity to claudin-4, allowing the efficient binding of PspA-C-CPE to NALT epithelium, including M cells.

### Nasal immunization with PspA-C-CPE efficiently induces PspA-specific antibody responses in both the respiratory and systemic compartments

**Fig 3:**
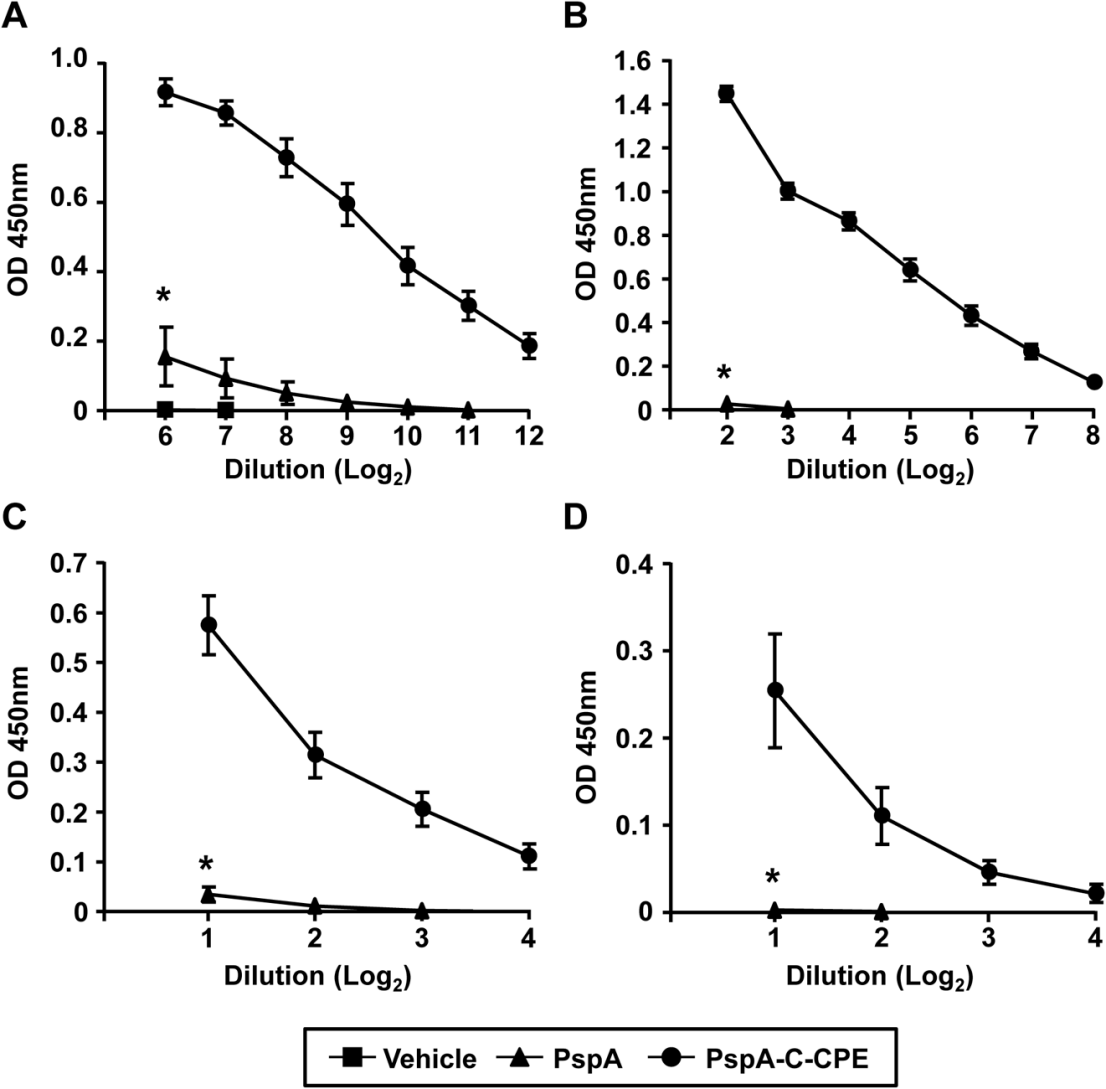
Induction of PspA-specific systemic and respiratory antibody responses by intranasal immunization with PspA-C-CPE. Mice were nasally immunized with vehicle, PspA alone, or PspA-C-CPE (PspA; 5 μg) once weekly for 3 weeks. One week after the last immunization, PspA-specific serum IgG (A), nasal IgA (B), BALF IgG (C), and IgA (D) were measured by ELISA. Data are shown as mean ± SEM and are representative of two independent experiments. Vehicle, n = 4; PspA, n = 5; PspA-C-CPE, n = 5. Values were compared by using the non-parametric Mann–Whitney *U* test. *P < 0.01.

Based on the efficient delivery of vaccine antigen to nasal epithlium including NALT by the use of PspA-C-CPE, our next experiment was aimed to investigate the PspA-specific immune responses induced by nasal immunization with PspA-C-CPE. In this study, mice were nasally immunized with mock, PspA alone, or PspA-C-CPE once a week for 3 weeks. One week after the last immunization, serum and respiratory samples (nasal wash and BALF) were collected for ELISA analysis to measure the production of PspA-specific antibodies. Mice nasally immunized with PspA-C-CPE showed higher levels of PspA-specific serum IgG (Fig 3A). In addition to systemic immune compartment, PspA-specific IgA responses were induced in the nasal washes (Fig 3B). Furthermore, mice nasally immunized with PspA-C-CPE showed higher levels of PspA-specific IgA and IgG responses in the BALF (Fig 3C and 3D). These findings indicate that the efficient delivery of PspA by nasal immunization using C-CPE was coincident with the induction of strong PspA-specific antibody responses in the respiratory tract and systemic immune compartments.

### Nasal immunization with PspA-C-CPE induces protective immunity against *S*. *pneumoniae* infection

**Fig 4:**
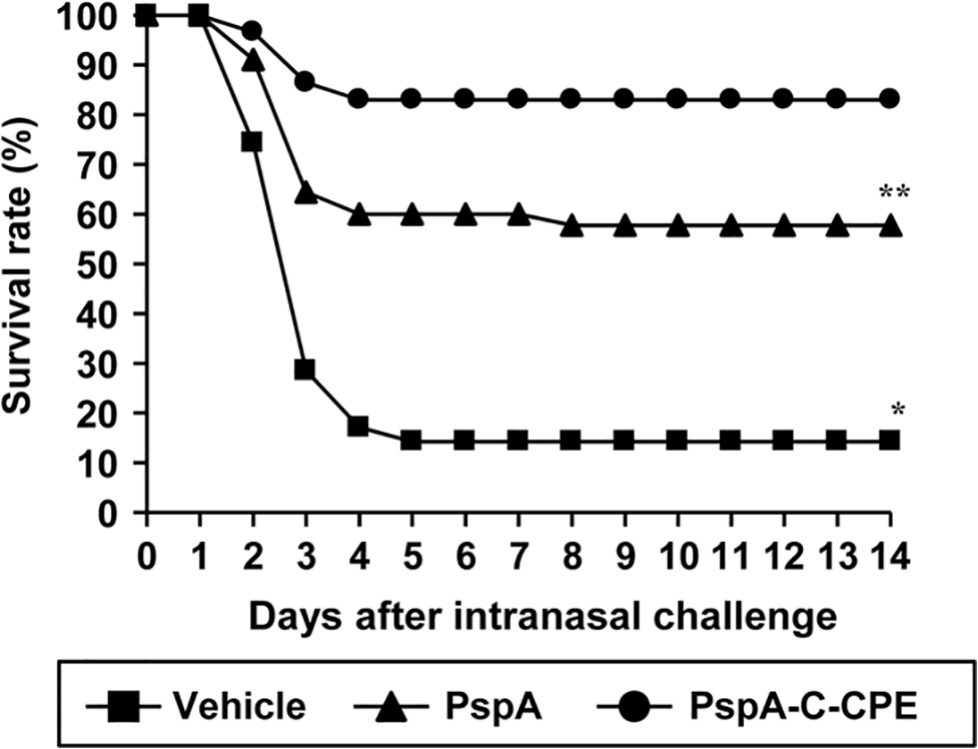
PspA-C-CPE-mediated induction of protective immunity against pneumococcal infection. Mice were nasally immunized with vehicle, PspA alone, or PspA-C-CPE (PspA; 5μg) once weekly for 3 weeks. One week after the last immunization, mice were intrarespiratory challenged with *S*. *pneumoniae* (5.0 × 10^6^ CFU/mouse), and their survival was monitored for 14 days. Survival was compared between groups by using the non-parametric Mann–Whitney *U* test. **P < 0.05, *P < 0.01. Data were collected four experiments. Vehicle, n = 35; PspA, n = 45; PspA-C-CPE, n = 59.

Finally, we evaluated whether the PspA-specific immune response induced by nasal immunization with PspA-C-CPE was sufficient to protect against pneumococcal infection. One week after the last immunization, mice underwent respiratory challenge with *S*. *pneumoniae* (5 × 10^6^ CFU/mouse). We monitored the survival rate until 14 days after infection. Few mice could survive after pneumococcal infection when they were nasally immunized with mock (less than 15%) or PspA alone (less than 60%) (Fig 4). In contrast, more than 80% of the mice survived in the same condition when mice received nasal immunization with PspA-C-CPE (Fig 4).

IL-17 is known to play important roles in the clearance of pneumococcal infection [29]. Thus, it is possible that IL-17-type innate immunity induced by C-CPE provides protective immunity against pneumococcal infection. However, we found that C-CPE alone did not induce IL-17-producing cells in the nasal passage and lung (S1 and S2 Figs). Consequently, mice receiving nasal administration of C-CPE alone could not survive after pneumococcal infection (S3 Fig). Thus, C-CPE alone did not induce IL-17-type innate immunity, which was insufficient to protect against pneumococcal infection. These findings indicate that nasal immunization with PspA-C-CPE induced PspA-specific acquired immunity, which was required for the protection against pneumococcal infection.

## Discussion

In this study, we showed that claudin-4-targeting using C-CPE elicited PspA-specific systemic and respiratory antibody responses that were sufficient to induce protection against pneumococcal infection. Various approaches for a vaccine delivery system have been reported. M cells have been shown to be potential target cells for vaccine delivery to MALTs. Some pathogens (e.g., *Salmonella*, reovirus, *Yersinia*, *E*. *coli*) use M cells as an invasion site, and the underlying molecular mechanisms including the ligand and receptor have been revealed [9, 30–32]. For instance, σ1, a surface protein of reovirus, binds to α2,3-sialic acid on M cells to invade its host [33]. FimH expressed on enterobacteria acts as a ligand for glycoprotein 2 for the organism’s invasion through M cells [32]. CPE binds claudin-4 which is expressed in intestinal mucosa [34] to show their pathogenesis. It was reported that claudin-4 is also expressed on antigen-sampling M cells in the NALT and GALT [28]. Thus, claudin-4 is a potent target to deliver vaccine to M cells. Our current study extend microbe-based vaccine delivery to MALT by showing the ability of C-CPE to bind to respiratory epithelial cells, including NALT M cells.

Professional antigen-sampling cells, M cells are covered by short villi and less densed mucus, and thus physically and chemically accessible to mucosally- administered antigen from the lumen of respiratory and intestinal tracts [3]. Basement of M cells form a pocket structure where dendritic cells are located [3], allowing efficient antigen transport to the dendritic cells. Then, dendritic cells present antigen to underlining T cells and B cells for the initation of antigen-specific immune responses [4]. Our current study demonstrated that PspA-C-CPE bound to M cells, which resulted in the internalization of antigen into NALT for the induction of PspA-specific antibody responses. In addition, we found that PspA-C-CPE bound to not only M cells but also NALT epithelium (Fig 2B), which might further enhace the antigen deposition into NALT. To this end, our previous study showed that C-CPE reversibly opens tight junctions and enhances mucosal absorption [35]. Therefore, C-CPE likely opens the tight junctions, allowing the fused antigen uptake through the epithelial cell layer. Moreover, claudin-4 contains a clathrin-sorting signal sequence in its C-terminal intracellular region [36, 37], leading to the possibility that PspA-C-CPE is taken up by epithelial cells via clathrin-dependent endocytosis.

Regarding the safety of a PspA-C-CPE-based vaccine, the parent CPE protein is known to causes food poisoning by binding to claudin-expressing intestinal epithelium [34]. CPE has two domains: the N-terminal domain contains the toxic function, whereas the C-terminal domain has the claudin-binding function. In the food poisoning mechanism of CPE, claudin-4-CPE complexes on intestinal cells oligomerize through their N-terminal domains, subsequently leading to alternated membranes permeability and cell death [34]. Because C-CPE lacks the toxic N- terminal domain of CPE [38, 39], C-CPE likely is non-toxic. We found normal levels of aspartate transaminase, alanine aminotransferase, and urea nitrogen in the blood of mice nasally immunized with C-CPE [40]. Therefore, a nasal vaccine delivery system using C-CPE is a safe and effective tool for the development of mucosal vaccines.

Currently, polysaccharide-based vaccine is approved for use as a pneumococcal vaccine in humans. However, this vaccine only induces serotype-specific immune responses. In contrast, we used PspA as a vaccine antigen in this study because PspA is highly antigenic and induces cross-activity among various pneumococcal strains [23, 41, 42]. PspA-specific IgA responses purportedly prevent colonization or the initial step of invasion of *S*. *pneumoniae* [43]. In addition, PspA-specific serum IgG responses are also important for the elimination of invasing *S*. *pneumoniae* [26]. PspA has an ability to interact with and fix complement component C3 and inhibit its deposition. Indeed, PspA-negative *S*. *pneumoniae* is immediately cleared from blood because of their inability to inhibit complement function [44]. Therefore, it is plausible that PspA-specific serum IgG prevents the PspA-mediated inhibition of complement function and facilitates bacterial elimination in a complement-dependent manner. Although the exact immunologic function of PspA-specific IgA in the respiratory tract remains unknown, surface choline-binding proteins, including PspA, are required for colonization of the nasal cavity [45]. Our current study demonstrated that nasal immunization with PspA-C-CPE induces PspA-specific serum IgG responses and respiratory IgA responses (Fig 3A–3D). Consequently, PspA-C-CPE likely prevents initial invasion by *S*. *pneumoniae* in the respiratory tract and lethal pathogenesis in the systemic compartments. Although PspA was wildly cross-reactive to various pneumococcal isolate, the distinct degrees of cross-reactivity were reported [41, 46]. In this issue, one possibile strategy for the improvement is to use PspA derived from selective clades (e.g., PspA4 and PspA5) for the high degree of cross reactivity [42]. Further, additional pneumococcal antigens such as pneumococcal surface adhesion A and pneumolysin can be combined for the strong protection against pneumococcal infection [47, 48].

In summary, we genetically prepared C-CPE fused with PspA and confirmed that PspA-C-CPE efficiently bound to NALT epithelium, including M cells. These functions led to the induction of PspA-specific antibody responses in both the systemic compartment and respiratory tract; these responses were sufficient to convey protection against pneumococcal infection. These findings suggest that C-CPE is an effective nasal vaccine delivery system for protection against pneumococcal infection.

## Supporting Information

S1 Fig C-CPE alone did not induce IL-17-producing cells at the nasal passage. Mice were nasally immunized with vehicle or C-CPE alone (2 μg) once weekly for 3 weeks. One week after the last immunization, nasal passage were collected for measurement of IL-17-produceing cells. Bar is median. Data are representative of two independent experiments (n = 5 for each experiment).
(TIF)

S2 Fig C-CPE alone did not induce IL-17-producing cells at the lung. Mice were nasally immunized with vehicle or C-CPE alone (2 μg) once weekly for 3 weeks. One week after the last immunization, lung were collected for measurement of IL-17-produceing cells. Bar is median. Data are representative of two independent experiments (n = 5 for each experiment).
(TIF)

S3 Fig C-CPE alone could not provide the protective immunity against pneumococcal infection. Mice were nasally immunized with vehicle or C-CPE alone (2 μg) once weekly for 3 weeks. One week after the last immunization, mice were intrarespiratory challenged with *S*. *pneumoniae* (5.0 × 10^6^ CFU/mouse), and their survival was monitored for 14 days. Data are representative of two independent experiments (n = 10 for each experiment).
(TIF)
