# Supplementary figures and images for: C-Terminal Clostridium perfringens Enterotoxin-Mediated Antigen Delivery for Nasal Pneumococcal Vaccine
Source: PLoS One. 2015 May 27;10(5):e0126352. doi: 10.1371/journal.pone.0126352 (PMC4446347; doi:10.1371/journal.pone.0126352)

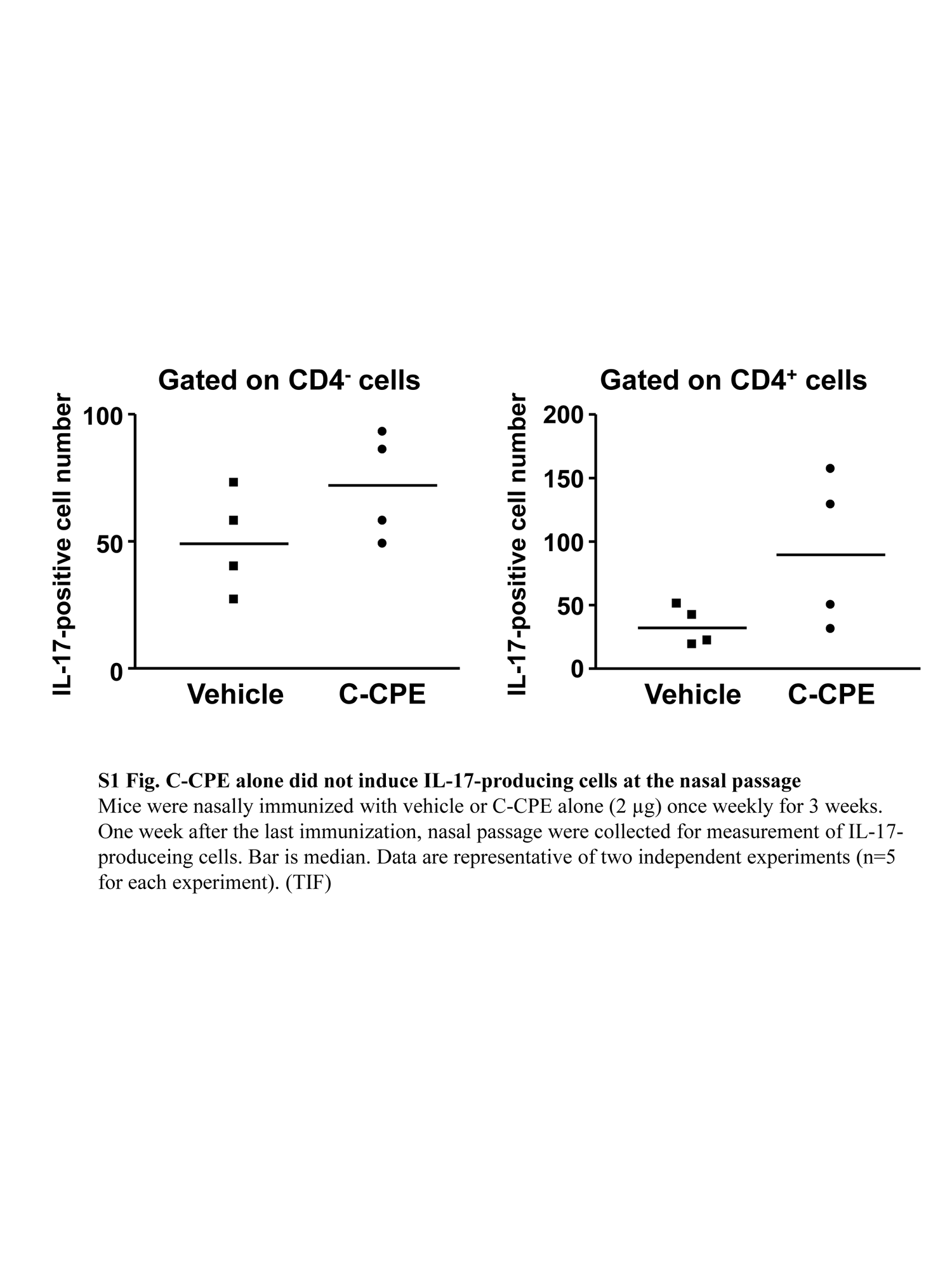

Supplement: S1 Fig — Mice were nasally immunized with vehicle or C-CPE alone (2 μg) once weekly for 3 weeks. One week after the last immunization, nasal passage were collected for measurement of IL-17-produceing cells. Bar is median. Data are representative of two independent experiments (n = 5 for each experiment). (TIF) [file pone.0126352.s001.tif]

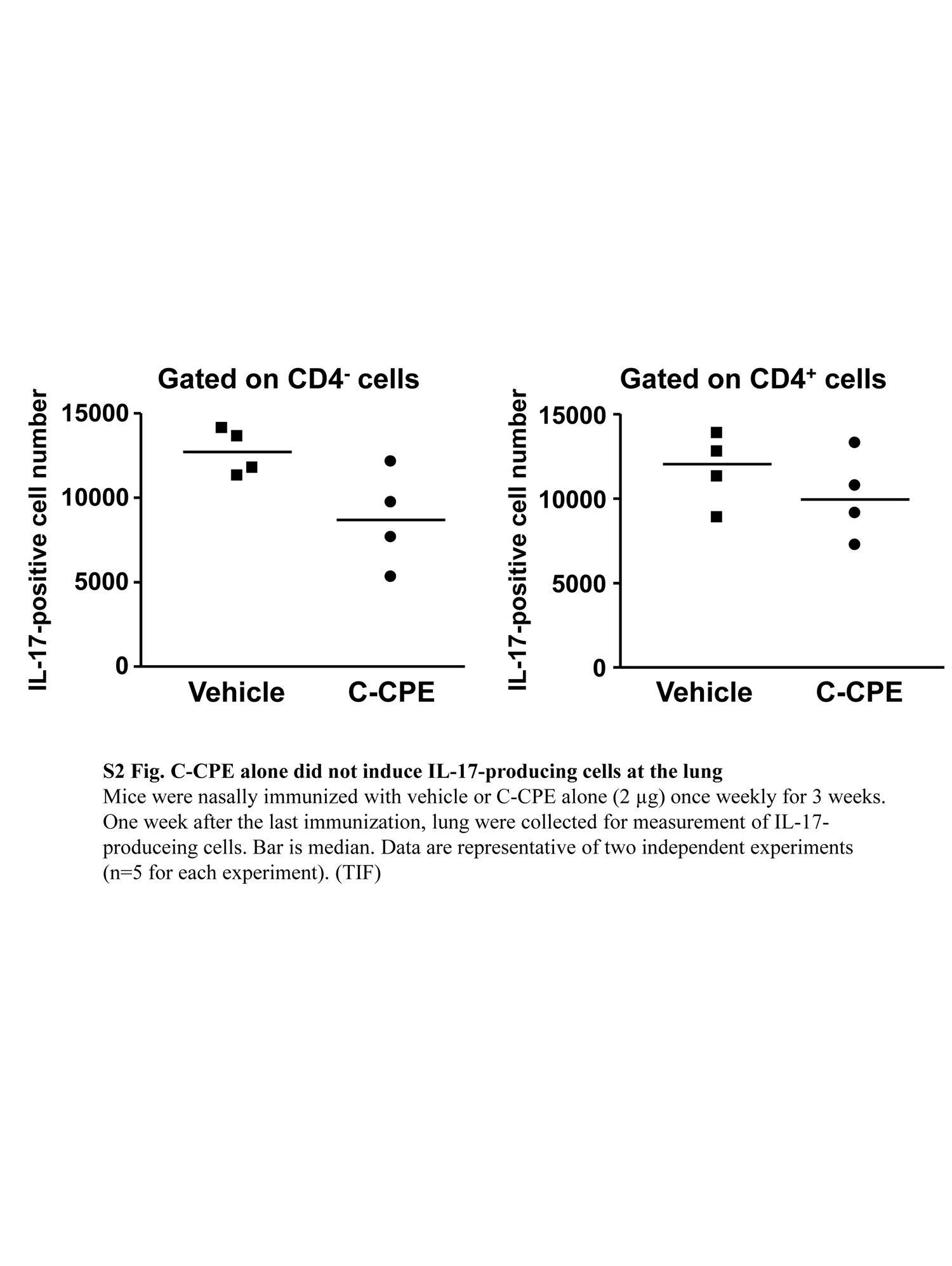

Supplement: S2 Fig — Mice were nasally immunized with vehicle or C-CPE alone (2 μg) once weekly for 3 weeks. One week after the last immunization, lung were collected for measurement of IL-17-produceing cells. Bar is median. Data are representative of two independent experiments (n = 5 for each experiment). (TIF) [file pone.0126352.s002.tif]

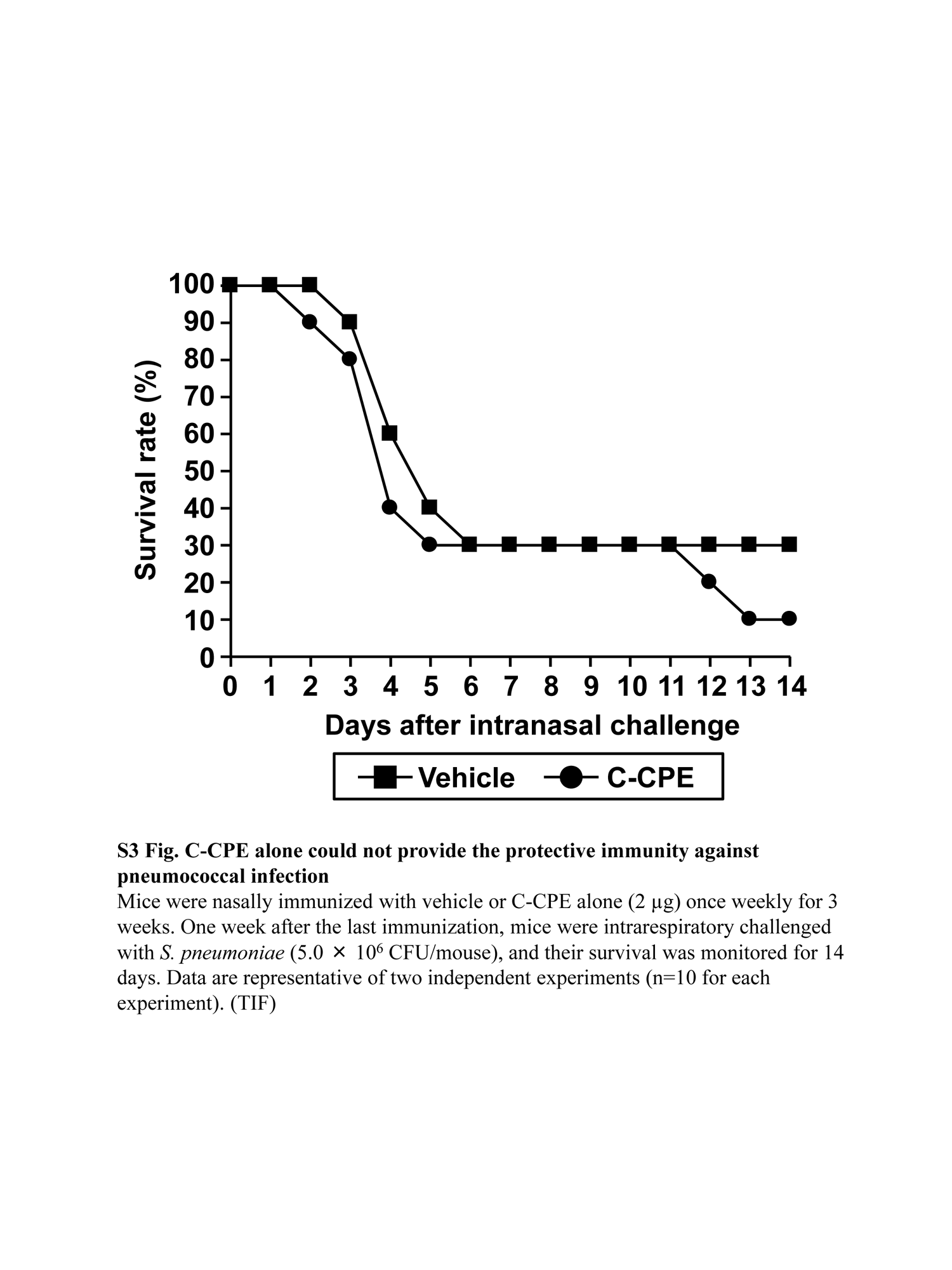

Supplement: S3 Fig — Mice were nasally immunized with vehicle or C-CPE alone (2 μg) once weekly for 3 weeks. One week after the last immunization, mice were intrarespiratory challenged with S. pneumoniae (5.0 × 10^6^ CFU/mouse), and their survival was monitored for 14 days. Data are representative of two independent experiments (n = 10 for each experiment). (TIF) [file pone.0126352.s003.tif]
